# Potential diagnostic markers of olanzapine efficiency for acute psychosis: a focus on peripheral biogenic amines

**DOI:** 10.1186/s12888-017-1562-1

**Published:** 2017-12-08

**Authors:** A. E. Taraskina, R. F. Nasyrova, A. M. Zabotina, D. N. Sosin, К. А. Sosina, E. E. Ershov, M. N. Grunina, E. M. Krupitsky

**Affiliations:** 1 Department of Addictions, Department of personalized psychiatry and neurology, V.M. Bekhterev National Medical Research Center Psychiatry and Neurology, ul. Bekhterev, d. 3, Saint-Petersburg, 192019 Russia; 2 Laboratory of Molecular Biology, First Saint Petersburg Pavlov State Medical University, L’va Tolstogo str. 6/8, Saint-Petersburg, 197022 Russia; 3 Laboratory of Molecular Human Genetics, National Research Centre “Kurchatov Institute”, Petersburg Nuclear Physics Institute named after B.P. Konstantinov, Leningrad district, Orlova Roscha, Leningrad district, Gatchina, 188300 Russia; 4 Saint Petersburg Psychiatric Hospital no. 1 named after P.P. Kashchenko, Leningrad region, district, s. Nikolskoye, ul. Menkovskaya, d. 10, Gatchina, Russia

**Keywords:** Acute psychosis, Schizophrenia, Antipsychotic treatment, Olanzapine, Peripheral blood mononuclear cells, Dopamine D4 receptor, Serotonin 2A (5-HT_2A_) receptor, Serum dopamine levels

## Abstract

**Background:**

Biomarkers are now widely used in many fields of medicine, and the identification of biomarkers that predict antipsychotic efficacy and adverse reactions is a growing area of psychiatric research. Monoamine molecules of the peripheral bloodstream are possible prospective biomarkers based on a growing body of evidence indicating that they may reflect specific changes in neurotransmitters in the brain. The aim of this study was to detect peripheral biogenic amine indicators of patients with acute psychosis and to test the correlations between the biological measures studied and the psychopathological status of the patients.

**Methods:**

This research included 60 patients with acute psychosis treated with olanzapine (*n* = 30) or haloperidol (*n* = 30). Here, we measured biogenic amine indicators, including mRNA levels of dopamine receptor D4 (*DRD4*) and the serotonin 2A receptor (*5HTR2A*), in peripheral blood mononuclear cells (PBMCs) using quantitative real-time polymerase chain reaction and serum dopamine concentrations by enzyme linked immunosorbent assay (ELISA). Psychopathological status was evaluated using psychometric scales. The assessments were conducted prior to and after 14 and 28 days of treatment.

**Results:**

The administration of haloperidol, but not olanzapine, up-regulated *5HTR2A* mRNA in a linear manner, albeit without statistical significance (*p* = 0.052). Both drugs had non-significant effects on *DRD4* mRNA levels. Nevertheless, a positive correlation was found between *DRD4* and *5HTR2A* mRNA levels over a longitudinal trajectory, suggesting co-expression of the two genes.

A significant positive correlation was observed between *5HTR2A* mRNA levels and total Positive and Negative Syndrome Scale (PANSS) scores in both groups of patients before treatment. A significant correlation between baseline *5HTR2A* mRNA levels and PANSS scores on days 14 and 28 of treatment remained for patients treated with olanzapine only. Moreover, a significant positive correlation was observed between blood serum dopamine levels and scores on extrapyramidal symptom scales in the olanzapine group.

**Conclusions:**

The *DRD4* and *5HTR2A* genes are co-expressed in PBMCs during antipsychotic administration. Despite a correlation between the studied biogenic amine indicators and the psychopathological status of patients, reliable biomarkers of treatment response could not be determined.

## Background

Schizophrenia is the most severe and prevalent disease among serious psychiatric disorders and is characterized by disturbances in cognitive, emotional and motor processes, affecting approximately 1% of the world’s population [1]. The etiology and pathophysiology of schizophrenia remain unclear. Several studies have suggested dopamine system changes in schizophrenia. The dopaminergic hypothesis for schizophrenia is still largely based on the consequences of pharmacologic manipulations of dopamine transmission by either mimicking or reducing the symptoms of schizophrenia [2, 3]. This “dopamine hyperfunction hypothesis” is the dominant theory in schizophrenia and has been supported by molecular, pharmacological and clinical evidence for more than 40 years [4, 5].

Pharmacotherapy for psychiatric disorders is one of the most difficult areas of psychiatry. Dopaminergic drugs (antipsychotics) are the drugs of choice for the pharmacological treatment of schizophrenia. Since the introduction of the first antipsychotic, chlorpromazine, in 1952, these drugs have constantly been improved. However, despite the continuous development of antipsychotics, inefficiency related to negative symptoms is frequent, and adverse effects (including extrapyramidal symptoms (EPS)) complicate therapy, even in modern second-generation antipsychotics [6, 7]. Therefore, identifying biomarkers that can predict treatment response in patients with schizophrenia will be an important step towards providing personalized medicine that will increase the efficiency and safety of therapy [8, 9].

Findings from studies using brain tissue biomarkers have not yet been translated into clinical use because brain biopsies are not accessible, neuroimaging techniques are expensive and the results are inconclusive. Therefore, in recent years, peripheral biomarkers for schizophrenia have been investigated as a valid alternative [10].

The original discovery of endogenous dopamine in human peripheral blood mononuclear cells (PBMCs) was made by Bergquist et al. [11] in 1994. PBMCs are an attractive tool; evidence indicates that lymphocytes can be used as biomarkers in the field of psychiatry because they may reveal the condition of cells located in the brain. Despite several methodological and theoretical limitations, numerous findings have suggested that PBMCs may serve as a cellular tool to identify deranged neurotransmission in neuropsychiatric diseases and monitor the effects of pharmacological treatments [12].

Numerous studies have suggested that PBMCs can express biogenic amine receptors on their membranes [13–15]. Studies on the alterations of biogenic amines in PBMCs from schizophrenic patients have mostly shown alterations of the expression of D2-like receptors of dopamine (D2, D3 and D4). Dopamine receptor D4 is one of the main targets of both classical antipsychotic drugs and second-generation antipsychotic drugs. The human protein coded by *DRD4* is located at chromosome region 11p15.5, close to a cluster of imprinted genes that are involved in the formation of behavioural phenotypes. Moreover, mutations in the *DRD4* gene have been associated with various behavioural phenotypes, including acute psychosis and schizophrenia [16–18]. In addition, a meta-analysis that was based on more than 1000 association studies of schizophrenia identified 16 genes that were mostly biogenic amine-related, including dopamine receptor D4 [19].

Serotonin [5-hydroxytryptamine (5-HT)] is a monoamine compound derived from the amino acid tryptophan that modulates dopamine function. Based on the pharmacological profiles of second-generation antipsychotics, one of the most frequently studied genes in this domain is *5HTR2A*, which encodes the 5-HT_2A_ receptor. Indeed, compared with conventional antipsychotics, second-generation (or atypical) antipsychotic drugs have a higher affinity for this receptor than the dopamine D2-like receptors. The *5HTR2A* gene is located at chromosome region 13q14.2 in a locus that has been linked with schizophrenia but has shown conflicting results in association studies [20].

It was independently shown that abnormalities in plasma biogenic amine metabolism may reflect the development of mental diseases, such as acute psychosis, and responses to antipsychotic treatment [21].

Considering their close links with the pharmacological properties of antipsychotics, peripheral biogenic amine-related molecules may be good indicators for treatment response, but evidence to support this position is lacking. **The aim of this study** was to detect peripheral biogenic amine indicators (*DRD4* and *5HTR2A* mRNA levels and dopamine concentrations in the serum) of patients with acute pshychosis and to test the correlations between the diverse biological measures studied and the psychopathological status of patients.

## Methods

### Participants

We recruited 60 patients aged 18–53 years with a primary diagnosis of acute psychosis according to the F20.X ICD-10 criteria [22]: 35 patients with paranoid schizophrenia (F 20.0), 19 patients with acute polymorphic psychotic disorder with symptoms of schizophrenia (F 23.1) and 6 patients with different subtypes (complete information in Table 1). All patients were Caucasian and of Russian descent and were recruited and assessed between June 2014 and April 2016. All participants were drug-naïve, first-episode patients, excluding the possibility that alterations detected in gene expression levels could be attributed to medication rather than the disorder itself. The inclusion criteria were as follows: subjects who were (a) diagnosed with a spectrum disorder of acute psychosis, (b) drug-naïve, first-episode; (c) aged 18–55 years; (d) male; and (e) able to comprehend the procedure and aims of the study. The exclusion criteria were as follows: subjects who (a) were diagnosed with mental retardation (IQ ≤ 70); (b) had a past history of head trauma; (c) had a serious neurologic disorder (epilepsy, stroke, Parkinson’s disease or dementia); or (d) had a co-morbid diagnosis of substance abuse disorder.

**Table 1:** Demographic and clinical data

Demographic and clinical data^1^	Patient group treated with haloperidol (*n* = 30)	Patient group treated with olanzapine (*n* = 30)
Daily dose (Range) (mg/day)	19.8 ± 5.6 (2.5–30)	18.5 ± 3.9 (10–20)
Age (Range) (yrs)	29.4 ± 8.1 (19–53)	26.4 ± 6.19 (18–43)
BMI^2^ (Range) (kg/m^2^)	23.4 ± 3.76 (17–31.7)	22.6 ± 4.1 (16.2–35.3)
Acute psychosis:		
Paranoid schizophrenia	21 (70%)	14 (46.7%)
Acute polymorphic psychotic disorder with symptoms of schizophrenia	9 (30%)	10 (33.3%)
Simple type of schizophrenia	–	1 (3.3%)
Another type of schizophrenia	–	1 (3.3%)
Acute polymorphic psychotic disorder with symptoms of schizophrenia	–	2 (6.7%)
Acute schizophrenic disorder	–	2 (6.7%)

^1^All values are reported as the means ± standard deviation

^2^BMI – Body mass index was calculated as weight in kilograms divided by height squared in metres

After randomization, the patients were assigned to one of two treatment groups: olanzapine (*n* = 30) or haloperidol (*n* = 30) (control group). No other psychoactive medications were allowed. The daily dose, demographics and clinical characteristics of the subjects are listed in Table 1. No significant differences were found in age or baseline BMI between these two groups (*p* = 0.166, Student’s unpaired t-test, two-tailed).

The psychopathological status of patients (negative and positive symptoms) was evaluated using the Positive and Negative Syndrome Scale (PANSS) [23], Clinical Global Impression of Severity (CGI-S) and Clinical Global Impression of Improvement (CGI-I) [24]. Current extrapyramidal symptoms were assessed using the Simpson-Angus Scale (SAS) [25] and the Extrapyramidal Rating Scale (EPRS).

*Ethics statement*. This study was conducted according to the principles expressed in the Declaration of Helsinki. Written informed consent was obtained from all patients participating in the study, and all procedures were approved by a local ethics committee.

All assessments were conducted at three points: before treatment (V1) and at days 14 and 28 post-administration of the antipsychotic (V2, V3).

### Blood sample collection

Blood samples of all subjects included in the study at three points of the examination (V1, V2 and V3) were collected. Twenty mL of fasting ulnar vein blood was drawn from each subject. Ten mL were collected in vacutainer tubes containing 0.5 mM ethylene di-amine tetra-acetic acid (EDTA), and ten mL were collected in vacutainer tubes without anticoagulant. All blood samples were taken from the left arm vein in the sitting position. Tubes were transferred to the lab in a flask within two hours.

### Serum separation

The whole blood samples from vacutainer tubes without anticoagulant were fractionated by centrifugation at 2000 r/min for 10 min at room temperature as soon as they were delivered to the laboratory. After centrifugation, serum was separated into the upper layer and then individually transferred to a clean tube. All serum samples were immediately stored at −80 °C.

### Peripheral blood mononuclear cell (PBMC) separation

PBMC were isolated from the blood in vacutainer tubes containing EDTA by standard density centrifugation (2500 rpm at 25 °C for 30 min without a break) using Ficoll-Paque PLUS (d = 1.077, GE Healthcare Biosciences). Then, the cells were washed with phosphate-buffered saline (PBS) and centrifuged. Plaques were frozen at −80 °C for further use.

### RNA extraction, cDNA synthesis and quantitative real-time polymerase chain reaction (RT-PCR)

Extraction of total ribonucleic acid (RNA) was performed on PBMCs using the «RNeasy Mini Kit» kit (Qiagen, Germany) according to the manufacturer’s instructions.

Total RNA was reverse-transcribed into cDNA with the Fermentas cDNA synthesis kit (RevertAidTM, Fermentas, USA). The reaction contained total RNA, 5× Reaction Buffer, oligo (dT)_18_ primer, RiboLock RNase Inhibitor, dNTP Mix (10 mM), RevertAid H Minus M-MulV Reverse Transcriptase (200 U/μL) and nuclease-free water up to 20 μL. The RNA and primer mixture was denatured at 65 °C for 5 min before the RT reaction. The reaction condition was 42 °C for 60 min, and the reaction was terminated by heating at 70 °C for 5 min.

qRT-PCR for target genes was conducted on the Thermocycler C1000 with the CFX96 module (BIO-RAD, USA) using TaqMan probes. Every sample was tested using parallel detection 3 times to obtain the average Ct value. Differences in total RNA per reaction were normalized using *GNB2L1* (guanine nucleotide binding protein beta polypeptide 2-like) as an endogenous control and a calibrator (the sample loaded in the first three wells of the plate). Primer pairs and probes for *DRD4* and *GNB2L1* (guanine nucleotide binding protein beta polypeptide 2-like) were designed using Primer Express software, and the sequences of the primers and probes for *5HTR2A* were obtained from the literature [26]. All primers and TaqMan probes were synthesized by Syntol JSC (Moscow, Russia). The sequences are shown in Table 2.

**Table 2:** The primer and TaqMan probe sequences of *DRD4*, *5HTR2A* and *GNB2L1*

Shot gene name	Sequence (5′ – 3′)	Gene name
*DRD4*	Forward primer GGC CAT GGA CGT CAT GCT Reverse primer TGA TGG CGC ACA GGT TGA FAM -TGC ACC GCC TCC AT- RTQ1	Human D4 dopamine receptor
*5HTR2A*	Forward primer GCAAGATGCCAAGACAACAGATAA Reverse primer TCACACACAGCTCACCTTTTCAT FAM -TGGTTGCTCTAGGAAAGCAG- RTQ1	Human 5-hydroxytryptamine (serotonin) 2A receptor
*GNB2L1*	Forward primer GAA TAC CCT GGG TGT GTG CAA Reverse primer GGA CAС AAG ACA CCC ACT CTG A R6G - TAC ACT GTC CAG GAT GAG A - BHQ2	Human guanine nucleotide binding protein beta polypeptide 2-like

Real-time PCR was carried out with 1 μL of cDNA templates using 1.5 μL of pairs of primers, 2 μL of TaqMan probes (100 nM, target gene and *GNB2L1*), 10× Reaction Buffer, MgCl2, 10 mM dNTP Mix and Taq polymerase for a final reaction volume of 50 μL. The reaction conditions for all genes were 95 °C for 15 min for pre-denaturation, 94 °C for 15 s for denaturation, 60 °C for 30 s for annealing, 72 °C for 30 s for extension and cycling for 40 rounds.

mRNA levels of *DRD4* and *5HTR2A* were expressed in arbitrary units using the following formula: qr. = 2-∆∆Ct (∆∆Ct = ∆Ct Sample - ∆Ct calibrator, ∆Ct = Ct Target gene – Ct *GNB2L1*).

### Peripheral blood serum dopamine level evaluation

After thawing, 100 μL of serum was used for the assay. Peripheral blood serum dopamine levels of all subjects were tested using the Dopamine Research enzyme linked immunosorbent assay (ELISA) according to the manual’s instructions (LDN Labor Diagnostika Nord GmbH & Co. KG) for the quantitative determination of dopamine in various biological samples. The xMark microplate spectrophotometer (BIO-RAD) was used for dopamine determination in this step.

### Statistical analysis

The statistical analysis was performed using statistical software SPSS version 21 (IBM, USA). Continuous variables were assessed for normal distribution, and a one-way ANOVA was then used to evaluate the significance of the differences. For dichotomous variables, the Chi-square test was used. Because the data for mRNA levels and dopamine serum levels were not normally distributed, non-parametric tests were used. The Mann-Whitney (U) test was performed to compare *DRD4* and *5HTR2A* mRNA levels and dopamine serum levels between treatment groups (haloperidol vs. olanzapine). The comparison between repeated measures at the longitudinal trajectory of these indicators within one treatment group was performed using Friedman’s test. PANSS, CGI, SAS and EPRS scores were not continuous variables; thus, Spearman’s correlation analysis was used to analyse the relationship between the mRNA levels of the two target genes, dopamine serum levels and scale scores. Individual statistical tests are described in the figure legends. The significance levels were set at *p* < 0.05*, *p* < 0.01** and *p* < 0.001***.

## Results

### Dynamics of the mental state in patients during the administration of antipsychotics

Between June 2014 and April 2016, 60 schizophrenic patients with first-episode psychosis were randomly assigned to olanzapine treatment (*n* = 30) or haloperidol treatment (*n* = 30 as a comparison [control] group). These antipsychotic drugs were chosen due to their preferred use in treating first psychotic episodes, with haloperidol as the first choice and olanzapine the second choice [27]. The patients included in the study were screened to identify novel peripheral markers (based on biogenic amines) of the efficiency of olanzapine treatment for schizophrenia. The demographic and clinical data of the two groups of schizophrenic patients under antipsychotic therapy are summarized in Table 1.

Both treatments were well-tolerated. No clinically relevant changes in vital signs were observed in either group. The mental state dynamics of the patients with acute psychosis in the two groups during treatment are summarized in Table 3.

**Table 3:** Psychiatric status

Group of patients	Treated with haloperidol (*n* = 30)			Treated with olanzapine (*n* = 30)		
Visit number	V1	V2	V3	V1	V2	V3
PANSS Total scores (mean ± sd)	91.4 ± 13.6	79.6 ± 14.7	73.5 ± 17.3	87.4 ± 18.9	72.1 ± 19.0	63.8 ± 19.3
CGI-S scores (mean ± sd)	5.4 ± 0.8	4.6 ± 0.9	4.04 ± 1.04	5.1 ± 0.9	4.14 ± 1.1	3.5 ± 1.1
CGI-I scores (mean ± sd)	–	3.0 ± 0.7	2.8 ± 0.9	–	2.89 ± 0.9	2.8 ± 1.1
SAS scores (mean ± sd)	0.17 ± 0.54	4.8 ± 4.14	6.6 ± 4.37	0.07 ± 0.26	0.96 ± 1.86	0.85 ± 1.5
ESRS scores (mean ± sd)	0.4 ± 1.42	18.1 ± 14.9	22.6 ± 14.9	0.45 ± 2.4	3.5 ± 5.3	3.2 ± 5.2

sd – standard deviation

PANSS – Positive and Negative Syndrome Scale for Schizophrenia

CGI-S – Clinical Global Impression of Severity

CGI-I – Clinical Global Impression of Improvement

SAS – Simpson-Angus Scale for Extrapyramidal Symptoms

ESRS – Extrapyramidal Rating Scale

**Fig. 1:**
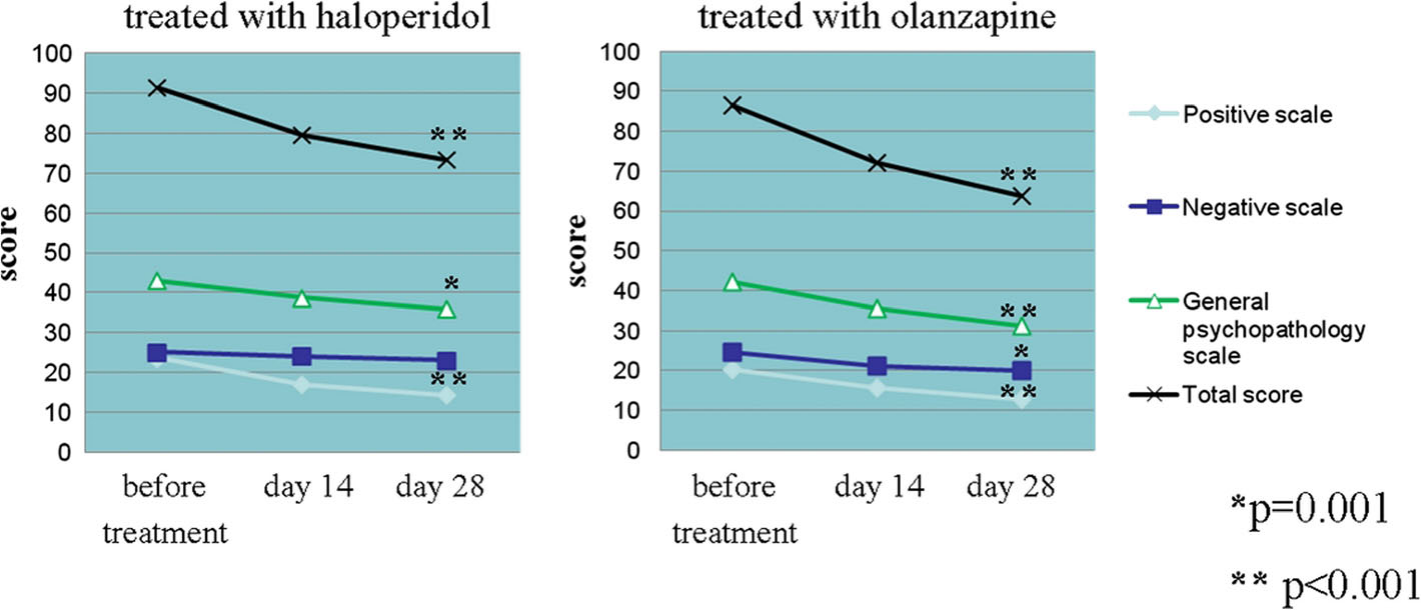
Dynamics of the mean total scores according to the PANSS scale

At baseline, the mean value of the total score according to the PANSS was 86.62 points for patients included in the group under olanzapine treatment and 91.36 points for the patients under haloperidol treatment (reliable differences were not observed). A positive response to treatment (a reduction in the total score according to the PANSS, *p*<0.001) was documented in both groups, regardless of the medication. Patients receiving olanzapine did not show greater improvement than patients receiving haloperidol relative to the PANSS scores on both day 14 (*p* = 0.549) and day 28 (*p* = 0.271) after the start of the therapy (comparison between treatment groups (haloperidol vs. olanzapine)). The reduction in the total score according to the PANSS was likely due to a reduction in the indices of positive symptoms and general psychopathological features for olanzapine and a reduction in the indices of positive symptoms for haloperidol. The data are shown in Fig. 1. Moreover, the mean values for the CGI-S and CGI-I scales decreased in both groups but were not significantly different between the two groups (*p* = 0.786).

Extrapyramidal side effects were more frequent in the haloperidol group than in the olanzapine group according to the SAS and ESRS. Twenty-seven (90%) patients on haloperidol had at least one extrapyramidal adverse event, compared with 18 (60%) patients on olanzapine (*p*<0.05). The spectrum of clinical manifestations in the two groups differed: parkinsonism and mixed extrapyramidal syndrome in the haloperidol group vs. neuroleptic tremor, drug parkinsonism and akathisia in the olanzapine group.

The results indicate that olanzapine and haloperidol are similarly effective and well-tolerated in routine treatment for a first psychotic episode associated with acute psychosis. However, haloperidol may produce more extrapyramidal adverse effects. Our results are consistent with global data [28, 29].

### Comparisons of *5HTR2A* and *DRD4* mRNA levels between both groups of patients during the administration of antipsychotics

**Fig. 2:**
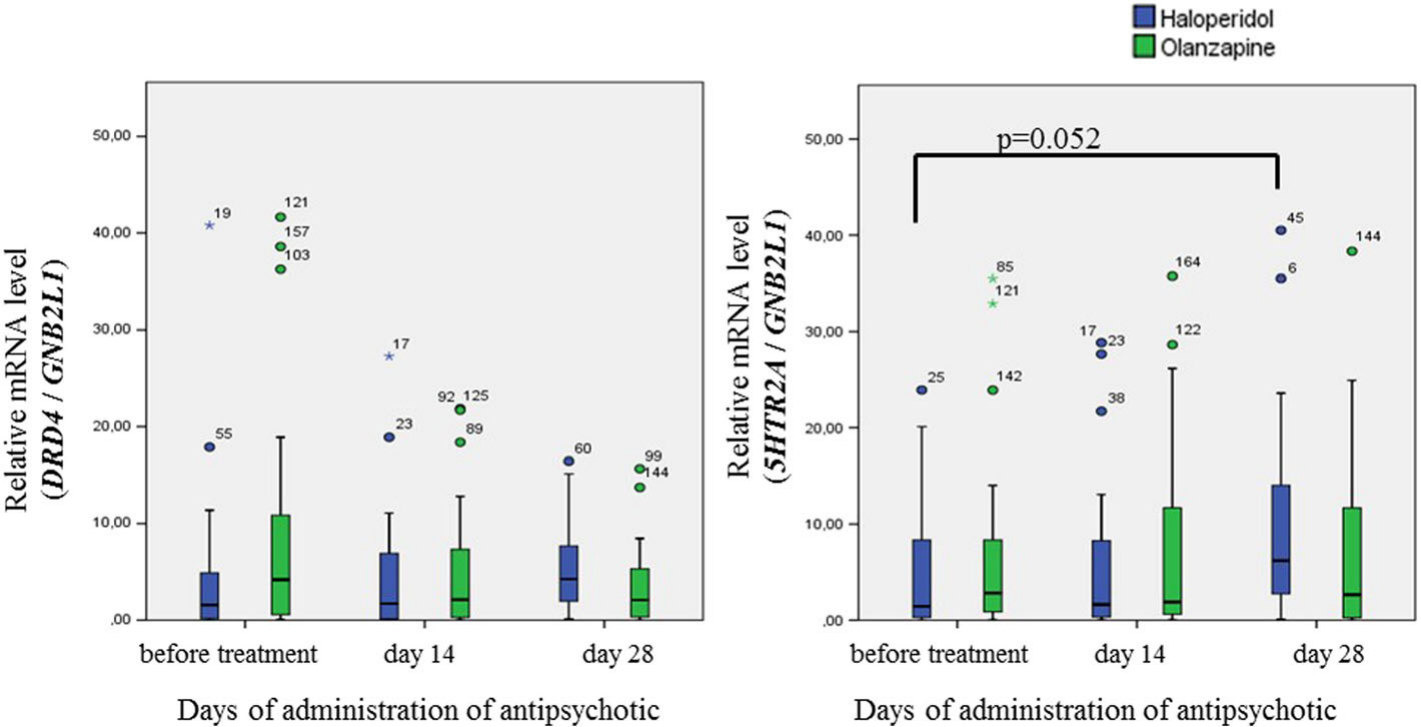
Expression levels of dopamine D4 receptor and 5-HT receptor subtype mRNA in PBMCs from schizophrenic patients during the administration of antipsychotics

The mRNA expression levels of *DRD4* and *5HTR2A* in PBMCs did not differ significantly between the patients on olanzapine and the controls (patients on haloperidol) before treatment (U = 277.5, *p* = 0.191 and U = 286.5 *p* = 0.085 for *DRD4* and *5HTR2A*, respectively). Friedman’s test for repeated measures showed that the mRNA expression level of *5HTR2A* in the PBMCs of patients receiving haloperidol increased slightly at V1 (before treatment), V2 (14 days of administration of the antipsychotic drug) and V3 (28 days of administration of the antipsychotic drug) (non-significant level, *p* = 0.052), but minimal effects on *5HTR2A* mRNA levels were demonstrated by olanzapine administration (*p* = 0.698). No significant differences were found in *DRD4* mRNA levels between the groups of patients during the administration of antipsychotics (*p* = 0.229 and *p* = 0.882 for patients receiving haloperidol and patients receiving olanzapine, respectively) (Fig. 2).

**Fig. 3:**
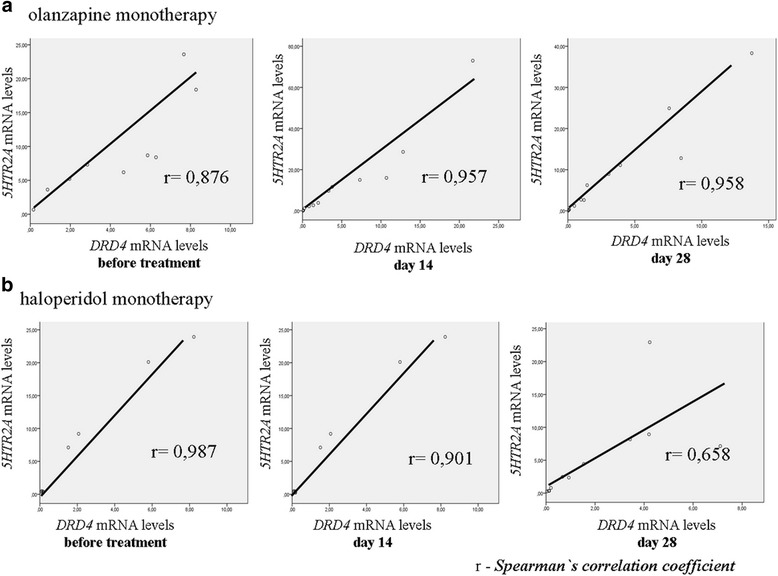
Association between *5HTR2A* and *DRD4* mRNA levels during the administration of antipsychotics (olanzapine (**a**) and haloperidol (**b**) monotherapy) in schizophrenic patients before treatment and at day 14 and 28 post-administration. Spearman’s correlation analysis was chosen to identify the significant association between the mRNA levels of *5HTR2A* and *DRD4*, which was significant at the level of 0.01

Therapy with haloperidol and olanzapine affected expression levels. However, co-expression of the two studied genes was observed. A significant association (positive correlation) was found between *DRD4* and *5HTR2A* mRNA levels at the three points of observation (*r* = 0.876, *p* < 0.0001; *r* = 0.957, *p* < 0.0001; and *r* = 0.958, *p* < 0.0001 for patients on olanzapine and *r* = 0.987, *p* < 0.0001; *r* = 0.901, *p* < 0.0001; and *r* = 0.658, *p* = 0.001 for patients on haloperidol, for V1, V2 and V3, respectively) (Fig. 3).

### Comparisons of dopamine serum levels between groups of patients during the administration of antipsychotics

The average concentration of peripheral dopamine at baseline among all patients included in the study was 110.8 ± 51.6 pg/mL (mean ± 3 SD) (range, from 637.3 to 9.1 pg/mL) under physiologically normal levels of dopamine in blood plasma (10–100 pg/mL), exceeding the threshold of the physiological concentration of peripheral dopamine. In some patients, the threshold was exceeded several times. The coefficient of variation (CV) for the internal analysis (intra assay) was 4.67, 11.38, 2.65 and 8.19 for four analysed plates, respectively. Inter assay variation (to monitor the precision of the results between different assays) was 2.65.

**Fig. 4:**
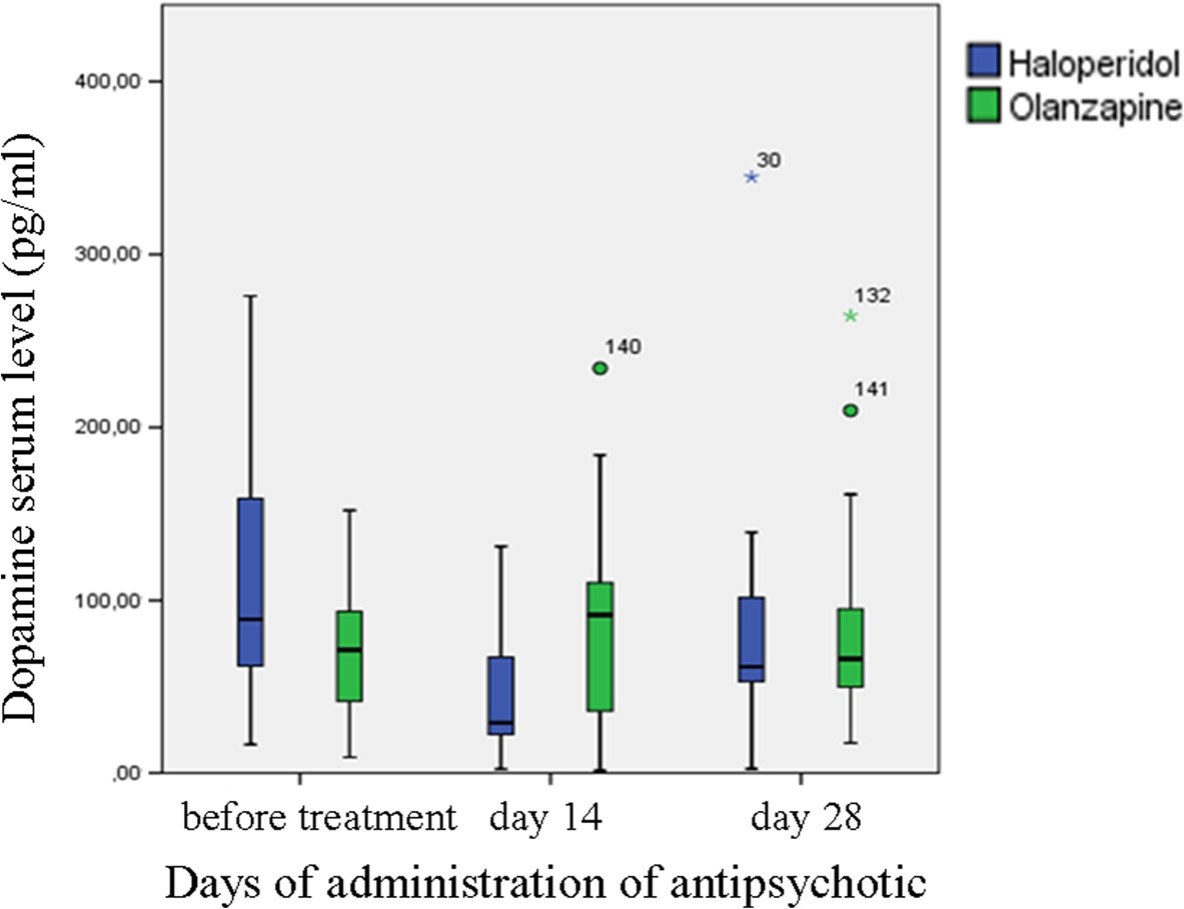
Dopamine serum levels from schizophrenic patients during the administration of antipsychotics

Friedman’s test for repeated measures did not reveal statistically reliable differences in the serum level of dopamine between visits (*p* = 0.568 and *p* = 0.396 for patients receiving haloperidol and patients receiving olanzapine, respectively) (Fig. 4). According to the averaged data, the patients with acute psychosis treated with antipsychotics (both olanzapine and haloperidol) had decreased levels of dopamine in the blood serum by day 14, indicating a positive response to therapy. However, by day 28 of therapy, the level of dopamine increased, correlating with the development of extrapyramidal symptoms.

### Correlation analysis between peripheral biogenic amine indicators (*DRD4* and *5HTR2A* mRNA levels and the dopamine concentration in serum) and scores on psychometric scales in patients with schizophrenia during the administration of antipsychotics

The correlation analysis showed that *5HTR2A* mRNA levels were significantly positively associated with total scores on the PANSS in patients with schizophrenia before treatment in both groups (*r* = 0.524, *p* = 0.020 and *r* = 0.653, *p* < 0.001 for patients on haloperidol and patients on olanzapine, respectively), reflecting the severity of disease. Against the background of antipsychotic therapy, a significant positive correlation between primary (before treatment) *5HTR2A* mRNA levels and total scores on the PANSS remained only for patients receiving olanzapine (*r* = 0.686, *p* < 0.001 and *r* = 0.715, *p* < 0.001, V2 and V3, respectively). For patients receiving haloperidol, this correlation was not established. Furthermore, the primary *5HTR2A* mRNA level was positively correlated with CGI-S scores in patients on olanzapine (Table 4). This finding can be explained by olanzapine’s affinity for the 5-HT_2A_ receptor, while haloperidol antagonizes only dopamine receptors. Despite a correlation between *5HTR2A* mRNA levels and scores on psychometric scales, reliable biomarkers of treatment response could not be determined.

**Table 4:** Spearman rank correlation analysis between *5HTR2A* mRNA levels and scores on psychometric scales in patients with schizophrenic disorder during the administration of antipsychotics

Group of patients			Treated with haloperidol	Treated with olanzapine
Indicator of peripheral biogenic amine			*5HTR2A* mRNA levels Before treatment	*5HTR2A* mRNA levels Before treatment
Scores of psychometric scales:			0.524*	0.653**
Administration of antipsychotic	Before treatment	Total PANSS		
		Positive PANSS	0.114	0.035
		Negative PANSS	0.237	0.329
		General psychopathology PANSS	0.239	0.217
		CGI-S	0.030	0.033
	Day 14	Total PANSS	−0.018	0.686**
		Positive PANSS	0.033	0.323
		Negative PANSS	0.080	0.332
		General psychopathology PANSS	0.011	0.379*
		CGI-S	0.067	0.338
	Day 28	Total PANSS	0.061	0.715**
		Positive PANSS	−0.092	0.466*
		Negative PANSS	−0.031	0.411*
		General psychopathology PANSS	0.146	0.392*
		CGI-S	−0.095	0.402*

*p < 0.05, **p < 0.001

In the patients with acute psychosis in the two pharmacotherapy groups, no significant association was found between the *DRD4* mRNA levels and PANSS scores or other scores.

A significant positive correlation was observed between the blood serum dopamine level and scores on extrapyramidal symptom scales in patients with schizophrenic disorder during the administration of olanzapine according to Spearman’s correlation test (Table 5).

**Table 5:** Spearman rank correlation analysis between the blood serum dopamine level and scores on extrapyramidal symptom scales in patients with schizophrenic disorder during the administration of antipsychotics

Indicator of peripheral biogenic amine			Blood serum dopamine level			Blood serum dopamine level		
Group of patients			Treated with haloperidol			Treated with olanzapine		
Administration of antipsychotic			Before treatment	Day 14	Day 28	Before treatment	Day 14	Day 28
Scores on the extrapyramidal symptom scales:			−0.149			−0.315		
Administration of antipsychotic	Before treatment	SAS						
		ESRS	−0.500			−0.316		
	Day 14	SAS		0.292			−0.465*	
		ESRS		0.175			−0.475*	
	Day 28	SAS			0.037			−0.609**
		ESRS			0.076			−0.609**

*p < 0.05, **p < 0.001

## Discussion

Schizophrenia is the most severe and prevalent disease among the acute psychoses. Motivated by the evidence that dopaminergic signalling in the central nervous system (CNS) plays a key role in the pathogenesis of the illness, reflecting the main target of antipsychotics, we tested the hypothesis that protein and gene expression levels of biogenic amines (dopamine and serotonin) may predict individual responses to drugs and the adverse reactions of every patient. Our primary targets were receptor genes, which represent only a part of the neurotransmitter system [19]. This effect is not limited to the CNS. Therefore, antipsychotic drugs may change the profiles of peripheral monoamine-related receptors and metabolites. Moreover, the results from several studies support the hypothesis that PBMCs may constitute a useful tool for investigating changes in the monoaminergic system in CNS pathologies and monitoring the consequences of pharmacological manipulations of biogenic amine transmission [10, 30, 31]. However, evidence is lacking for this hypothesis, as the study on changes in peripheral monoamine-related molecules is limited.

The current research was undertaken to explore peripheral blood-based biomarkers that could distinguish individuals with respect to their clinical response to antipsychotic treatment with olanzapine. Olanzapine’s efficacy in acute psychosis is mediated through dopamine and serotonin type 2A antagonism and involves the normalization of dopamine levels. Therefore, we studied the mRNA levels of the *DRD4* and *5HTR2A* genes in PBMCs and dopamine concentrations in the serum.

According to the results of our study, therapy with haloperidol and olanzapine affected *DRD4* and *5HTR2A* mRNA levels, and these changes were dynamic and linear. Furthermore, we are the first to show the co-expression of dopamine and serotonin receptors (both are types of biogenic amine receptors), proving the existence of interactions between monoamine neurotransmitter pathways. 5-HT_2A_ receptors modulate dopamine function and thereby influence dopamine-dependent responses and the co-expression of the examined genes, which stabilize neurotransmission.

Previous studies of monoamine neurotransmitter system alterations in schizophrenic patients’ PBMCs have mostly targeted easily detectable peripheral molecular markers to diagnose psychiatric disorders. There is consistent evidence to support the possibility of identifying changes in the expression of different dopamine receptor subtypes in PBMCs from schizophrenic patients [12, 32]. However, in the vast majority of studies, indicators of the biogenic amines were examined only before the beginning of therapy. Studies in which indicators of the biogenic amines were examined during treatment are rare and do not consider the full range of both monoamine-related molecules and antipsychotic drugs used in psychiatry. Nevertheless, lymphocyte mRNA levels of several monoamine receptors were reported to show earlier dynamic, linear changes in people with schizophrenia during treatment with antipsychotics [33].

Although Friedman’s test for repeated measures did not show statistically significant differences in the mRNA expression levels of *5HTR2A* or *DRD4* in PBMCs during treatment with both antipsychotics, the administration of haloperidol showed a tendency to up-regulate *5HTR2A* mRNA in a linear manner (non-significant level). This finding can be explained by the different affinity profiles of the antipsychotics included in the study. Haloperidol acts only via dopamine receptor antagonism, decreasing only D4 receptor transmission, but does not influence serotonin transmission. Olanzapine is a multiple receptor antagonist with a low affinity for dopamine receptors and a higher affinity for 5HT_2A_ receptors. These two drugs appear to alter the functional activity of the genes encoding target proteins. We believe that the typical one-directional-action antipsychotics cause an imbalance of individual physiological levels of biogenic amines, which is highlighted by a decreasing correlation relative to the co-expression of various monoamines in our results. The drugs cause increased gene expression, but protein levels are not influenced by these drugs.

The gene expression and protein levels of the studied receptors showed extensive individual variation in patients before treatment (data not shown). This finding is consistent with results concerning the various biogenic amine markers (gene expression and protein levels) reported by other authors and our own previous studies. Previously, we showed that it is possible to divide patients into groups according to their levels (high or low) of lymphocytes that carry different amounts of dopamine receptors on their cell membranes regardless of the pathological process, which also applies to healthy subjects. Furthermore, a positive correlation was found between the number of lymphocytes carrying different investigated subtypes of the D2-like receptors (*p* < 0.01) [34].

Few studies have analysed the relationship between biogenic amine-related protein mRNA levels in the PBMCs of schizophrenic patients and the psychiatric symptoms of the disease [18, 32, 35]. For example, Brito-Melo et al. revealed that the percentage of T-lymphocyte subsets (CD4 and CD8) carrying D4 receptors was increased in schizophrenic patients compared with that in controls. In that study, the Abnormal Involuntary Movement Scale (AIMS) scores were inversely related to D4-carrying cells, but the number of lymphocytes carrying D2 receptors was positively related to the PANSS, the AIMS and the Brief Psychiatric Rating Scale (BPRS) [36]. Here, we did not find a statistically significant correlation between *DRD4* gene expression levels and the psychiatric symptoms estimated by the PANSS before therapy or during antipsychotic administration. Our study differs from the aforementioned investigations because biogenic amine markers and scores on the psychometric scales were measured several times during therapy. According to the results of our study, the *DRD4* mRNA level could not be used as a predictor of the efficiency and safety of olanzapine or haloperidol therapy. Additionally, the *DRD2* gene requires further investigation. Our results do not contradict the use of *DRD4* mRNA levels as a marker of the predisposition to psychosis (schizotypy).

5-HT_2A_ has been one of the most studied serotonin receptors [37]. *HTR2A* gene allelic variants have been shown to affect gene expression, subsequently influencing olanzapine treatment response [38, 39]. Our results suggest that 5*HTR2A* expression levels in the PBMCs of patients with acute psychosis before treatment correlate with the clinical response to olanzapine. Unfortunately, these findings do not allow us to consider this indicator as a biomarker of treatment response. Although the identification of biomarkers that can be used as predictors of antipsychotic treatment response is not a new trend in psychiatry, several monoamine markers have not shown a strong association with either the response to antipsychotics or certain adverse effects.

The relationship between peripheral blood dopamine levels and the development of some neuropsychiatric disorders has been definitively proven. Abnormal peripheral blood dopamine levels are observed in patients with pathologies of brain neurotransmitter balance, such as addiction and neurogenic disorders [40, 41]. High dopamine concentrations were found in subjects with high scores on the psychometric scales. Furthermore, abnormalities in plasma monoamine metabolism partly reflect some symptoms of schizophrenia [21]. On one hand, we did not find a significant correlation between dopamine levels in blood serum and PANNS scores. However, the peripheral blood dopamine levels of the patients included in the study were higher than the physiological norm. We can conclude that a high peripheral blood dopamine level is associated with CNS disorders (e.g., acute psychosis and schizophrenia), but it is not a specific marker for mental dysfunction and has no relationship with the severity of the illness. On the other hand, there was a correlation between the Extrapyramidal Symptom Scale scores and blood dopamine levels during olanzapine administration from day 14 of therapy. EPS remain the most serious problem among schizophrenic patients, even in the era of new antipsychotics with less affinity toward D2 receptors [7]. Unfortunately, although we showed a correlation between blood dopamine levels and EPS development with olanzapine administration, dopamine levels cannot serve as a predictor of side effects. Interestingly, haloperidol therapy did not exhibit the relationships described above, even though the Extrapyramidal Symptom Scale scores were higher in this treatment group, possibly because of the different pathways of dopamine synthesis and dopamine release in the CNS and blood as compensatory responses to the blockade of another range of receptors by haloperidol compared with olanzapine.

Several limitations of the present study need to be mentioned. One limitation of our study is the small sample size as we recruited only drug-naïve, first-episode patients with acute psychosis treated with one of two antipsychotic drugs in monotherapy. The limited sample size diminishes the power of the study. Another critical issue of our study is the short-term follow-up of the participants (4-week treatment). Longer monitoring of mRNA levels during antipsychotic administration can potentially yield more significant results. Moreover, although the presence of a serious neurologic disorder was an exclusion criterion, we cannot exclude the possibility that the studied patients had some somatic and psychiatric comorbidities.

It is well-known that the analysis of amine receptors in PBMCs is useful for evaluating the functional properties of the monoamines that underlie variations in complex psychological and psychopathological traits. However, some limitations to the use of PBMCs as possible biomarkers for studying changes in the monoamine system in neuropsychiatric disease should be acknowledged. In different environments, these cells are subject to different regulatory mechanisms. Another important limitation of this study was that our survey did not include all blood markers of biogenic amines (e.g., other olanzapine-targeted receptors). Further studies with more patients are necessary to investigate these variables. Moreover, the 5*HTR2A* mRNA level may serve as a diagnostic biomarker of therapeutic efficacy, but clear criteria are required relative to its range in groups of patients with different olanzapine therapy outcomes.

## Conclusions

Approaches in which patients are divided into subgroups according to their genetically determined response to antipsychotics and side effects of treatment are very promising. Once biomarker-based therapeutics are established, they can guide treatment selection. In the future, knowledge оf the genetic predispositions to various responses to antipsychotic treatment will provide an opportunity for clinicians to implement personalized medicine in the mental health arena. The use of easily detectable peripheral molecular markers can substantially facilitate the diagnosis of psychiatric disorders and improve treatment efficacy.

Based on global publications, many studies have aimed to identify peripheral biomarkers of acute psychosis, with schizophrenia as the first objective. However, only a few studies have reported the discovery of clinically relevant, discriminating prognostic markers that show clinically significant sensitivity and specificity in the predisposition of medical outcomes.

According to our results, the 5*HTR2A* expression level in the PBMCs of patients with acute psychosis before treatment correlates with the clinical response to olanzapine. Unfortunately, these findings do not allow us to consider this indicator as a biomarker of treatment response. However, the results of the present study may provide a potential implication for future diagnostics in treatment outcomes and for the development of personalized antipsychotic therapy.

Taken together, the implementation of personalized medicine approaches to schizophrenia treatment is in the early stage. Prospective trials evaluating the predictive value and clinical utility of established prognostic markers are required to expand the use of current research findings in clinical practice. We are confident that the information obtained from such prospective trials will facilitate the further clinical implementation of marker-predictors, presenting revolutionary changes in schizophrenia treatment in the near future.

## Abbreviations

5HTR2A: Serotonin 2A receptor
AIMS: Abnormal Involuntary Movement Scale
BMI: Body mass index
BPRS: Brief Psychiatric Rating Scale
CGI-I: Clinical Global Impression of Improvement
CGI-S: Clinical Global Impression of Severity
CNS: Central nervous system
DRD3: Dopamine receptor D3
DRD4: Dopamine receptor D4
EPRS: Extrapyramidal Rating Scale
EPS: Extrapyramidal symptoms
GNB2L1: Guanine nucleotide binding protein beta polypeptide 2-like
PANSS: Positive and Negative Syndrome Scale
PBMCs: Peripheral blood mononuclear cells
SAS: Simpson-Angus Scale
SNP: Single nucleotide polymorphism
